# Selected Problems of Random Free Vibrations of Rectangular Thin Plates with Viscoelastic Dampers

**DOI:** 10.3390/ma15196811

**Published:** 2022-09-30

**Authors:** Marcin Kamiński, Agnieszka Lenartowicz, Michał Guminiak, Maciej Przychodzki

**Affiliations:** 1 Department of Structural Mechanics, Faculty of Civil Engineering, Architecture & Environmental Engineering, Lodz University of Technology, Al. Politechniki 6, 90-924 Lodz, Poland; 2 Doctoral School, Poznan University of Technology, Piotrowo 3 Street, 60-965 Poznan, Poland; 3 Department of Structural Mechanics, Institute of Structural Analysis, Poznan University of Technology, 60-965 Poznan, Poland

**Keywords:** finite element method, free damped vibrations, Kirchhoff–Love plates, viscoelastic dampers, continuation method, semi-analytical probabilistic technique, stochastic perturbation technique, Monte Carlo simulations

## Abstract

The main motivation of this work was to present a semi-analytical extension of the correspondence principle in stochastic dynamics. It is demonstrated for the stochastic structural free vibrations of Kirchhoff–Love elastic, isotropic and rectangular plates supported by viscoelastic generalized Maxwell dampers. The ambient temperature of the plate affects the dampers only and is included in a mathematical model using the frequency–temperature correspondence principle. The free vibration problem of the plate–viscoelastic damper system is solved using the continuation method and also the Finite Element Method (FEM). The stochastic approach begins with an initial deterministic sensitivity analysis to detect the most influential parameters and numerical FEM recovery of the polynomial representation for lower eigenfrequencies versus these parameters. A final symbolic integration leads to the first four basic probabilistic characteristics, all delivered as functions of the input uncertainties.

## 1. Introduction

Probabilistic mechanics is a topic that has been extensively studied, e.g., in [1,2], and one of its fundamental numerical methods—the Stochastic Finite Element Method (SFEM)—was invented and has been applied in the context of thin rectangular plate bending [3]. Considering multiple geometric scale uncertainties, the bending analysis of thin plates may also be performed using the Wavelet-based Stochastic Finite Element Method [4]. The SFEM was efficiently applied for the stochastic dynamic response analysis of graphite–epoxy composite plates [5]; other studies in this area can be found in [6,7]. An interesting scientific and engineering problem, the modeling of propeller blades, is presented in [8]. The authors did not use the typical random approach; however, they did perform an analysis of the deviation histogram of machining errors that may be random in nature.

The probabilistic structural response for the free damped vibrations of thin elastic and isotropic plates resting on viscoelastic supports is considered in this work. The nonlinear eigenproblem is solved here to determine the natural frequencies of the plate–viscoelastic damper system, and its solution is obtained thanks to the iterative continuation method presented by Lewandowski et al. [9,10,11]. The authors considered different types of viscoelastic dampers based on the generalized Maxwell model of a damper. An experimental study considering a generalized Maxwell model for nonlinear viscoelastic dampers was comprehensively performed by Lu et al. [12]. A comprehensive overview of some other deterministic and stochastic methods used in dynamics can be found in [13]. Several uncorrelated Gaussian random design variables are considered in this study, with an initial sensitivity study leading to the selection of the most influential parameters. The Least-Squares Method enables the determination of the random polynomials of eigenfrequencies, whose further integration with the Gaussian kernel finally returns the probabilistic characteristics. It should be underlined that the FEM experiments were entirely programmed in the Octave environment, whereas sensitivity and probabilistic analyses were all implemented in the computer algebra system MAPLE. The most important novelty of this work is the common application of a probabilistic numerical apparatus for a solution of an eigenproblem obtained using the continuation method.

## 2. Eigenvibration Analysis Methodology

**Figure 1:**
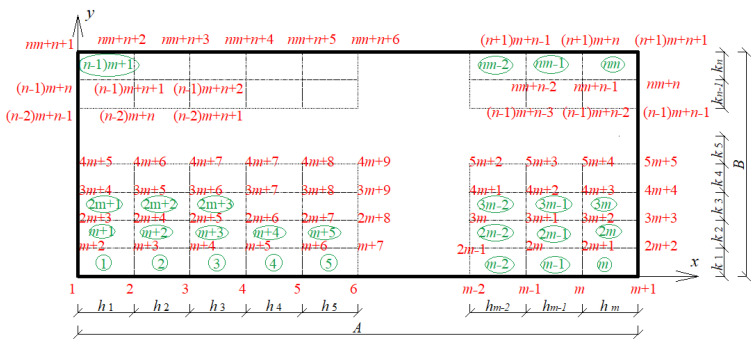
Plate discretization with rectangular plate finite elements.

**Figure 2:**
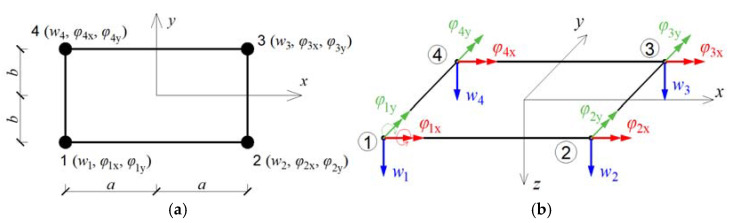
Finite element type plQ4 used for discretization of the tested plate: (**a**) node numbering; (**b**) active degrees of freedom. The numbers 1–4 are the element node numbers.

The main purpose of this analysis is to determine the first few natural frequencies of thin rectangular elastic and isotropic plates supported by viscoelastic dampers and also their first four probabilistic characteristics. The numerical analysis of this problem is based upon the Finite Element Method (FEM) with a regular discretization including 4-node-plate finite elements with linear approximation functions, as shown in Figure 1 and Figure 2 [14]. The deformation vector $w_{i}^{e}$ of the i-th node within the finite element e can be written as

(1) $$w_{i}^{e}={\left[\begin{array}{ccc} w_{i} & \varphi_{ix} & \varphi_{iy} \end{array}\right]}^{T}={\left[\begin{array}{ccc} w_{i} & \frac{\partial w_{i}}{\partial y} & -\frac{\partial w_{i}}{\partial x} \end{array}\right]}^{T};i=1,\;2,\;3,\;4.$$

The displacement field within the element e is expressed as a linear combination of shape functions $N_{k}^{e}\left(x,y\right)$:

(2) $$w^{e}\left(x,y\right)=N_{e}w_{e},$$

where $w_{e}={\left[w_{1}^{e},\;w_{2}^{e},\;w_{3}^{e},\;w_{4}^{e}\right]}^{T}$ and $N_{e}=\left[N_{1}^{e},\;N_{2}^{e},\;N_{3}^{e},\;\ldots N_{12}^{e}\right]$. The element stiffness matrix **K***e* and the consistent mass matrix $M_{e}$ are defined in the traditional way. The stiffness matrix is derived here analytically, and the mass matrix is derived numerically using 16-point Gaussian quadrature. Further, it is known that the equation of motion of a structure with viscoelastic dampers can be written in the following form [9,10]:

(3) $$M\ddot{q}\left(t\right)+C\dot{q}\left(t\right)+Kq\left(t\right)=f\left(t\right).$$

where C denotes the global plate-damping matrix.

The application of the Laplace transform with zero initial conditions leads to the following transform of Equation (3):

(4) $$\left(s^{2}M+sC+K\right)\bar{q}\left(s\right)=\bar{f}\left(s\right),$$

where $\bar{q}\left(s\right)$ is the ℒ-transform of q(t), and $\bar{f}\left(s\right)$ can be expressed as

(5) $$\bar{f}\left(s\right)=-\overset{n_{d}}{\underset{r=1}{\sum }}\left(K_{r}+G_{r}\left(s\right)\right)L_{r}\bar{q}\left(s\right).$$

**Figure 3:**
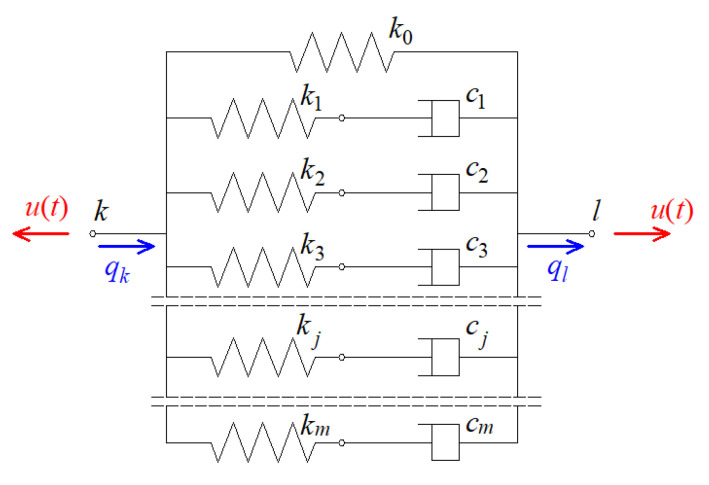
Generalized Maxwell model of viscoelastic damper.

In this formula, $n_{d}$ is the total number of dampers attached to the plate at selected nodes of a finite element mesh, and $L_{r}$ is a global matrix indicating the location of the r-th damper in the plate. A viscoelastic damper is represented graphically in Figure 3, and it consists of m spring-dashpot elements and an additional spring element. Each of the Maxwell elements contains a viscous part with the constant $c_{j}$ and an elastic part with the constant $k_{j}$, where j=1, 2,…,m.

All quantities appearing in Equation (5) can be expressed as follows:

(6) $$K_{r}=k_{0};\;G_{r}\left(s\right)=\overset{m}{\underset{j=1}{\sum }}\frac{k_{j}s}{\nu_{j}+s}$$

where $\nu_{j}=k_{j}/c_{j}$ is the quotient of the stiffness and damping coefficients of the j-th Maxwell element. Obviously, the stiffness and damping parameters, $k_{j}$ and $c_{j}$, of the individual elements constituting the viscoelastic damper attached to the structure additionally depend upon the temperature. Let the damper parameters be known for a certain reference temperature $T_{0}$ and have the values $k_{j}$ and $c_{j}$. The damper model constants at temperature T can be expressed using the frequency–temperature correspondence principle as [9,10]

(7) $${\bar{k}}_{j}=k_{j};j=0,\;1,\;2,\ldots ,m,$$

(8) $${\bar{c}}_{j}=c_{j}\alpha_{T};j=1,\;2,\ldots ,m.$$

The shift factor $\alpha_{T}$ is a function of temperature T and can be expressed by the William–Landel–Ferry formula as [15,16]

(9) $$\log_{10}\alpha_{T}=\frac{-C_{1}\left(T-T_{0}\right)}{C_{2}+T-T_{0}},$$

where $C_{1}$ and $C_{2}$ are experimental constants. After substituting Equation (5) into Equation (4), the ℒ-transform of Equation (3) of the motion of a plate with viscoelastic dampers takes the following form:

(10) $$\left(s^{2}M+sC+K+K_{d}+G_{d}\left(s\right)\right)\bar{q}\left(s\right)=0,$$

where

(11) $$K_{d}=\overset{n_{d}}{\underset{r=1}{\sum }}K_{r}L_{r},$$

(12) $$G_{d}\left(s\right)=\overset{n_{d}}{\underset{r=1}{\sum }}G_{r}\left(s\right)L_{r}.$$

Equation (10) represents a nonlinear eigenproblem that is solved for the eigenvalue s and the eigenvector $\bar{q}\left(s\right)$ using the continuation method comprehensively described by Lewandowski in, e.g., [9,10]. Below, the foundations of this method are quoted.

In the case of Equation (10), the components containing the variable s in the first power are multiplied by the parameter κ∈[0;1]. The equation can then be re-written as

(13) $$h_{1}\left(\bar{q},s\right)=D\left(s\right)\bar{q}\left(s\right)=0,$$

where

(14) $$D\left(s\right)=s^{2}M+\kappa sC+K+K_{d}+\kappa G_{d}\left(s\right)$$

In order for the elements of the eigenvector $\bar{q}$ corresponding to the eigenvalue s to be determined unambiguously, an additional normalizing equation of the following form is introduced into the matrix in Equation (13):

(15) $$h_{2}\left(\bar{q},s\right)=\frac{1}{2}\bar{q}{\left(s\right)}^{T}\frac{\partial D\left(s\right)}{\partial s}\bar{q}\left(s\right)-a=0,$$

where a has a given value.

In the first step of the continuation method, in Equation (13), the parameter $\kappa_{1}=0$ is assumed, and the generalized eigenproblem is solved.

(16) $$\left(s^{2}M+K+K_{d}\right)\bar{q}\left(s\right)=0$$

This problem was solved in the Octave program using the built-in command ‘eig’, which allows both standard and generalized eigenproblems to be solved. As a result of solving this problem, the first approximations of eigenvalues $s_{1}^{\left(1\right)},s_{2}^{\left(1\right)},\ldots ,s_{3n}^{\left(1\right)}$ and eigenvectors ${\bar{q}}_{1}^{\left(1\right)},{\bar{q}}_{2}^{\left(1\right)},\ldots ,{\bar{q}}_{3n}^{\left(1\right)}$ are obtained. On their basis, the parameter $a_{j}^{\left(1\right)}=s_{j}^{\left(1\right)}{\left({\bar{q}}_{j}^{\left(1\right)}\right)}^{T}M{\bar{q}}_{j}^{\left(1\right)}$, where j=1, 2,…, 3n, is determined.

In the *l*-th step (l=2, 3, 4,…), the increment $∆\kappa_{l}$ is assumed, and the Newton method is used to solve the system of Equation (13) with the additional Equation (15). For this purpose, the system of incremental equations of the Newton method is solved using $\kappa_{l}=\kappa_{l-1}+∆\kappa_{l}$, $s_{j}^{\left(k-1\right)}$, ${\bar{q}}_{j}^{\left(k-1\right)}$ and $a_{j}^{\left(k-1\right)}$. This system of equations takes the following form:

(17) $$\{\begin{array}{c} \frac{\partial h_{1}}{\partial \bar{q}}\delta \bar{q}+\frac{\partial h_{1}}{\partial s}\delta s=-h_{1},\\ \frac{\partial h_{2}}{\partial \bar{q}}\delta \bar{q}+\frac{\partial h_{2}}{\partial s}\delta s=-h_{2,} \end{array}$$

where

(18a) $$\frac{\partial h_{1}}{\partial \bar{q}}=D\left(s\right)=s^{2}M+\kappa sC+K+K_{d}+\kappa G_{d}\left(s\right),$$

(18b) $$\frac{\partial h_{1}}{\partial s}=\left(2sM+\kappa C+\kappa \frac{\partial G_{d}\left(s\right)}{\partial s}\right)\bar{q},$$

(18c) $$\frac{\partial h_{2}}{\partial \bar{q}}={\bar{q}}^{T}\left(2sM+\kappa C+\kappa \frac{\partial G_{d}\left(s\right)}{\partial s}\right),$$

(18d) $$\frac{\partial h_{2}}{\partial s}=\frac{1}{2}{\bar{q}}^{T}\left(2M+\kappa \frac{\partial^{2}G_{d}\left(s\right)}{\partial s^{2}}\right)$$

The derivatives in Equations (18b)–(18d) are calculated as follows:

(19a) $$\frac{\partial G_{d}\left(s\right)}{\partial s}=\overset{n_{d}}{\underset{r=1}{\sum }}\overset{m}{\underset{j=1}{\sum }}\frac{k_{j}\nu_{j}}{{\left(\nu_{j}+s\right)}^{2}}L_{r},$$

(19b) $$\frac{\partial^{2}G_{d}\left(s\right)}{\partial s^{2}}=\overset{n_{d}}{\underset{r=1}{\sum }}\overset{m}{\underset{j=1}{\sum }}-\frac{2k_{j}\nu_{j}}{{\left(\nu_{j}+s\right)}^{3}}L_{r.}$$

The increments $\delta \bar{q}$ and δs are obtained from the system of Equation (17), and the following are calculated:

(20a) $$s_{j}^{\left(k\right)}=s_{j}^{\left(k-1\right)}+\delta s,$$

(20b) $${\bar{q}}_{j}^{\left(k\right)}={\bar{q}}_{j}^{\left(k-1\right)}+\delta \bar{q},$$

(20c) $$a_{j}^{\left(k\right)}=\frac{1}{2}{\left({\bar{q}}_{j}^{\left(k\right)}\right)}^{T}\frac{\partial D\left(s\right)}{\partial s}{\bar{q}}_{j}^{\left(k\right)}.$$

Successive approximations of the j-th eigenvalue and the j-th eigenvector in the l-th step of the algorithm are calculated until the desired accuracies $\varepsilon_{1}$ and $\varepsilon_{2}$, of the final results are achieved, that is, until the following inequalities are satisfied:

(21) $$\delta s<\varepsilon_{1}\left|s_{j}^{\left(k\right)}\right|,$$

(22) $$\|\delta \bar{q}\|<\varepsilon_{2}\|{\bar{q}}_{j}^{\left(k\right)}\|.$$

The final values of $s_{j}^{\left(k\right)}$, ${\bar{q}}_{j}^{\left(k\right)}$ and $a_{j}^{\left(k\right)}$ obtained in the l-th step are taken as starting values for step l+1 and the new parameter $\kappa_{l+1}=\kappa_{l}+∆\kappa_{l+1}$.

The procedure described above is carried out up to the value of the parameter κ=1, when the final eigenvalues and eigenvectors for the nonlinear eigenproblem in Equation (10) are obtained.

The obtained eigenvalues of the problem in Equation (10) are complex numbers of the form $s_{j}=\mu_{j}+i\eta_{j}$. On this basis, the j-th natural frequency $\omega_{j}$ of the structure and the non-dimensional damping ratio $\gamma_{j}$ of the j-th mode of vibration are determined from the formulas:

(23) $$\omega_{j}^{2}=\mu_{j}^{2}+\eta_{j}^{2};\;\gamma_{j}=-\frac{\mu_{j}}{\omega_{j}}.$$

The continuation method makes it possible to calculate the first few natural frequencies and the corresponding non-dimensional damping ratios without having to solve the entire nonlinear eigenproblem. Using proprietary algorithms written in the Octave programming language, the natural frequencies of a plate equipped with viscoelastic vibration dampers are determined. In the program, the user can independently select the positions of the selected number of dampers in the FEM mesh nodes. With the help of proprietary software, the matrices occurring in the nonlinear eigenproblem in Equation (10) are determined. Using the built-in command, the program solves the generalized eigenproblem in Equation (16). Then, the program uses the above-described iterative algorithm of the continuation method, which allows the unknown natural frequencies of a plate equipped with viscoelastic vibration dampers to be determined.

## 3. Sensitivity and Uncertainty Analyses

The so-called normalized sensitivity gradients are determined using the following standard definition [1]:

(24) $$\frac{\Delta \omega_{i}}{\Delta v_{j}}\equiv {\left(\frac{\partial \omega_{i}}{\partial v_{j}}\right)}_{{\bar{v}}_{j}}\cdot \frac{{\bar{v}}_{j}}{{\bar{\omega }}_{i}}$$

where $\bar{v_{j}}$ denotes the mean value of the given parameter $v_{j}$. The following design parameters affecting the natural frequencies of the rectangular plate are checked at the initial stage: (*i*) geometric dimensions of the plate $l_{x}\times l_{y}\times H$, (*ii*) material constants of the plate E, $\nu_{p}$ and $\rho_{p}$, (*iii*) damper parameters $k_{0}$, $k_{1}$ and $c_{1}$, (*iv*) the ambient temperature of the plate, namely, T, and (*v*) the reference temperature $T_{0}$. Further probabilistic analysis is carried out for two parameters exhibiting the highest positive and negative sensitivity coefficients. The eigenfrequencies $\omega_{i}$ of the plate under consideration are all found via the polynomial basis

(25) $$\omega_{i}=\sum_{j=1}^{n}C_{ij}v^{j}$$

via the Least-Squares Method fittings made on the basis of several FEM solutions for varying values of the parameter *v* [1,2]. Statistical optimization of this basis order is employed through the common minimization of the fitting variance and the maximization of the correlation factor. Finally, the basic probabilistic characteristics, i.e., expected values, standard deviations, coefficients of variation, skewness and kurtosis, are computed. The following integral definitions are applied:

(26) $$E\left[\omega_{i}\right]=\int_{-\infty }^{+\infty }\sum_{j=1}^{n}C_{ij}v^{j}\;p_{v}(x)dx,\;\sigma \left(\omega_{i}\right)={\left\{\int_{-\infty }^{+\infty }\;{\left(\sum_{j=1}^{n}C_{ij}v^{j}-E\left[\omega_{i}\right]\right)}^{2}\;p_{v}(x)dx\right\}}^{\frac{1}{2}}$$

(27) $$\alpha \left(\omega_{i}\right)=\left|\frac{\sigma \left(\omega_{i}\right)}{E\left[\omega_{i}\right]}\right|,\;\beta \left(\omega_{i}\right)=\frac{\mu_{3}\left(\omega_{i}\right)}{\sigma^{3}\left(\omega_{i}\right)},\;\kappa \left(\omega_{i}\right)=\frac{\mu_{4}\left(\omega_{i}\right)}{\sigma^{4}\left(\omega_{i}\right)}$$

## 4. Numerical Experiment

**Figure 4:**
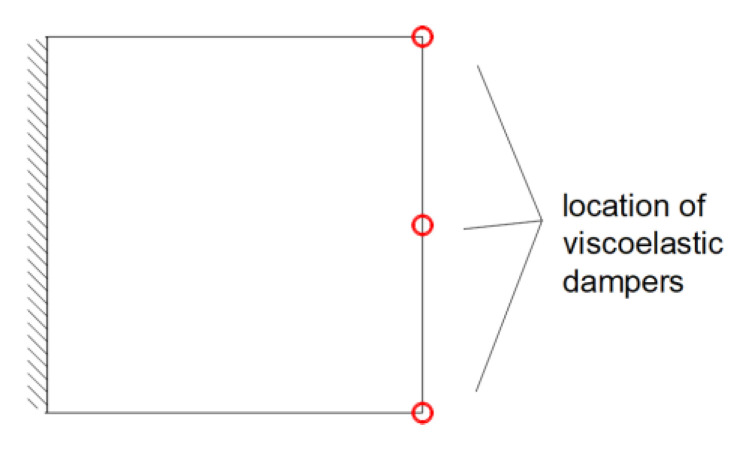
Plate fixed on one edge with three viscoelastic dampers.

A square isotropic plate fixed on one edge was discretized using the 14 × 14 plate rectangular finite element mesh, whose material properties are *E* = 205 GPa, *ν*_p_ = 0.3 and *ρ*_p_ = 7850 kg/m^3^. The plate dimensions are equal to $l_{x}\times l_{y}\times H=\left(2.0\times 2.0\times 0.01\right)\;m$. Three viscoelastic dampers are attached in the middle and at both ends of the free edge of the plate (see Figure 4).

**Figure 5:**
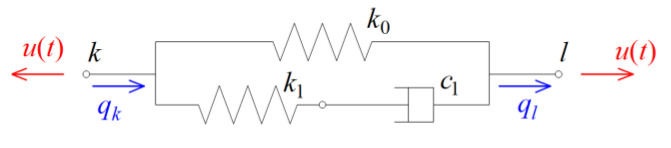
Model of a viscoelastic damper attached to the tested plate.

These dampers contain a single spring element and also a Maxwell element (Figure 5) with the following parameters at $T_{0}=0.2\;℃$: $k_{0}=108.56\;N/m;\;k_{1}=19968.09\;N/m;\;c_{1}=229.63\;\mathrm{Ns}/m.$

The influence of temperature on the values of the above-mentioned parameters is taken into account by applying the frequency–temperature correspondence principle. In order to calculate the value of the shift function from Equation (9), the values of the constants $C_{1}=19.5$ and $C_{2}=80.2$ were adopted. The initial sensitivity analysis results were computed analytically using polynomial responses and are compared in Table 1 below. Quite expectedly, the two most influential parameters for the given plate are its thickness (the minimum value of the gradient) and the edge length (the maximum gradient).

So, these two parameters were further selected for stochastic analysis. They were treated as Gaussian variables having expected values equal to the mean values given above and a coefficient of variation belonging to the interval α(v)∈[0.00, 0.20]. Such a wide interval was assumed to check theoretical variations in all characteristics being computed, and it includes the statistical scattering of all possible measurement techniques. It should be noted that the time effort and computer power required for the semi-analytical probabilistic solutions were only a little bit greater than the deterministic origin. Polynomial approximation was used to describe the response function. The degree of the polynomial was assumed to be 4 or 5 depending on the necessary accuracy of matching the response function. Examples of polynomials obtained for a random plate side length and a random plate thickness are given in Chapter 5 by the relations in (28) and (29), respectively.

**Figure 6:**
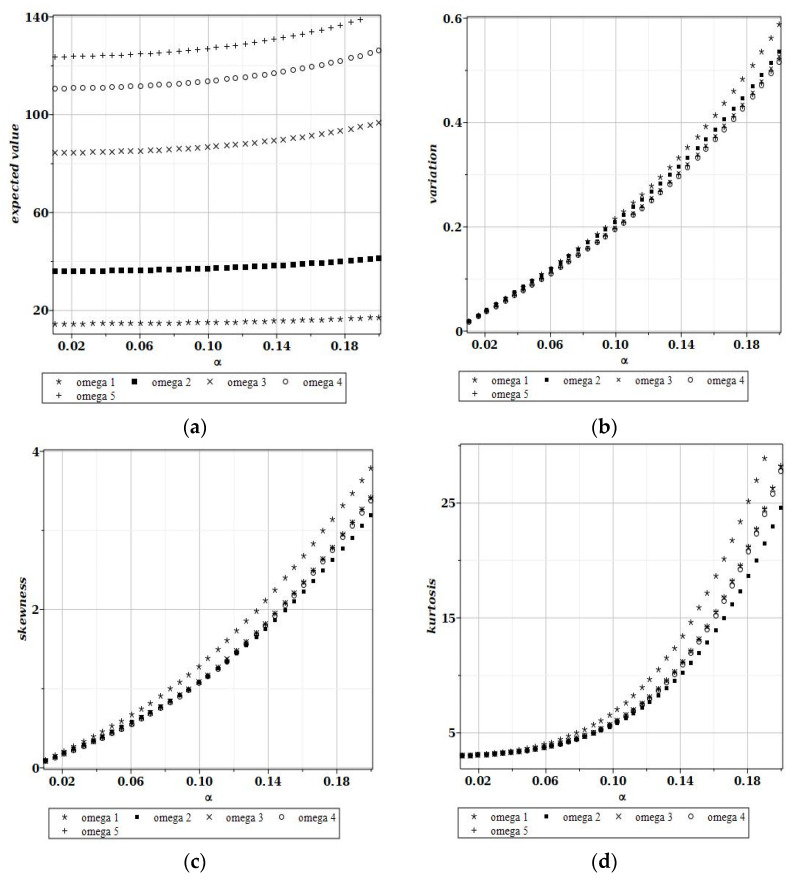
Expected values (**a**), coefficients of variation (**b**), skewness (**c**) and kurtosis (**d**) for a random length of the plate.

**Figure 7:**
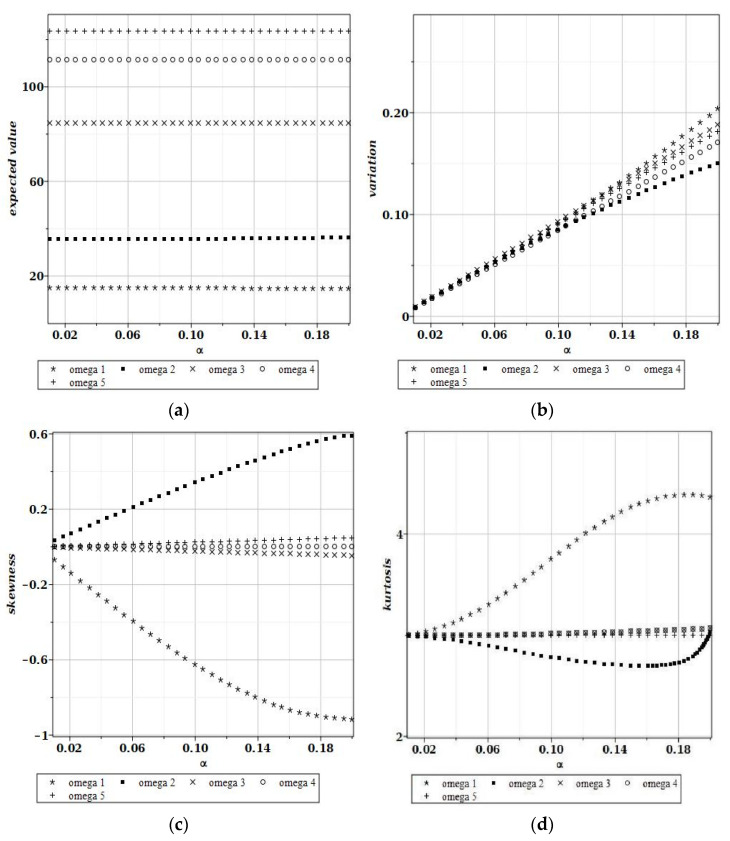
Expected values (**a**), coefficients of variation (**b**), skewness (**c**) and kurtosis (**d**) for a random thickness of the plate.

Figure 6 and Figure 7 show the graphs of the dependence of the expected value, variance, skewness and kurtosis on the coefficient of variance when the random variable is the plate side length and its thickness, respectively; each of the graphs in Figure 6a–d and Figure 7a–d shows these characteristics for the first five natural frequencies, $\omega_{1}–\omega_{5}$. These probabilistic coefficients were all found from their integral definitions, and they can be treated as exact in the probabilistic context (no convergence studies are necessary).

It is seen in Figure 6 that Gaussian uncertainty in the plate length causes some nonlinear increases even for the expected values of the fundamental free vibrations. This is in contradiction to the case illustrated in Figure 7, where they are simply constant. Moreover, the plate length randomness greatly amplifies the uncertainty in this problem, because its output-to-input ratio equals almost 3; the plate thickness shows a direct interrelation between the input and output CoVs (Figure 7b). Interestingly, the largest statistical dispersion is always associated with the first eigenvalue. Finally, it is seen that both the skewness and kurtosis monotonously increase together with an additional increase in the input CoV in Figure 6. Hence, the positive non-symmetry and concentration about the expected values remarkably increase together with the input uncertainty level. One notices that the differences between probabilistic characteristics for various eigenfrequencies are rather small. Figure 7c,d show that higher-order probabilistic coefficients relevant to the plate thickness oscillate about (for the 1st and the 2nd) or are almost equal to 0 (for higher eigenfrequencies).

## 5. Comparative Analysis—Results Validation

In order to better illustrate the previously obtained results, calculations were performed using three probabilistic approaches: the Semi-Analytical Method (SAM), the Stochastic Perturbation Technique (SPT) and Monte Carlo simulations (MCSs).

These analyses were applied for a plate exhibiting Gaussian uncertainty in the first natural frequency, uniquely defined by its mean value and the specific range of its coefficient of variation, i.e., α(b)∈[0.00, 0.025]. The global response function for random plate side length *l_x_* = *l_y_* = *l* was obtained in the form of the following fourth-order polynomial:

(28) $$\begin{array}{cc} \omega_{1}\left(l\right)= & 367.570938927759-555.768532731197\cdot l+336.646138548979\cdot l^{2}\;\\ & -92.8342487373826\cdot l^{3}+9.66595643939497\cdot l^{4} \end{array}$$

**Figure 8:**
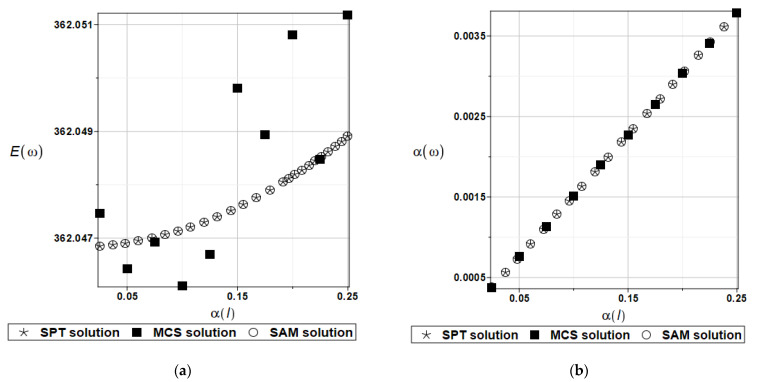

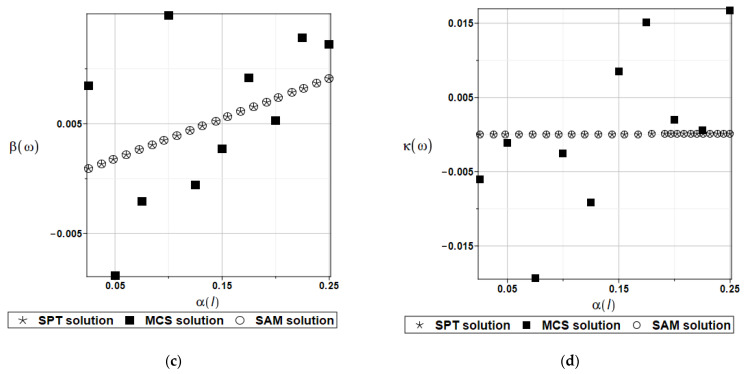
Expected values (**a**), coefficients of variation (**b**), skewness (**c**) and kurtosis (**d**) for a random plate side length.

The choice of the degree of the approximating polynomial to the random quantity was dictated by the sufficient accuracy of matching the response function. The number of trials for the Monte Carlo simulations was equal to 150,000. The expected values E(l), coefficients of variation α(l), skewness β(l) and kurtosis κ(l) of the first natural frequency are presented in turn in Figure 8. Considering the large variations in the resulting statistics, the expected values and coefficients of variation for the first natural frequency are presented in Table 2 and Table 3.

A very good convergence of the results of the SAM and SPT approaches can be observed. On the other hand, MCS results are in good agreement only for the coefficient of variations α(l).

Another random parameter for which the validation of the calculation results was performed is the plate thickness. In this case, the global response function for a random plate thickness *h* was obtained in the form of the following fifth-order polynomial:

(29) $$\begin{array}{cc} \omega_{1}\left(h\right)= & 8.35555733333377-7824.96232261055\cdot h\;\\ & +2.41798719696965\cdot 10^{6}\cdot h^{2}-2.55138948717944\cdot 10^{8}\cdot h^{3}\;\\ & +1.18596617132865\cdot 10^{10}\cdot h^{4}-2.02512179487178\cdot 10^{11}\cdot h^{5} \end{array}$$

**Figure 9:**
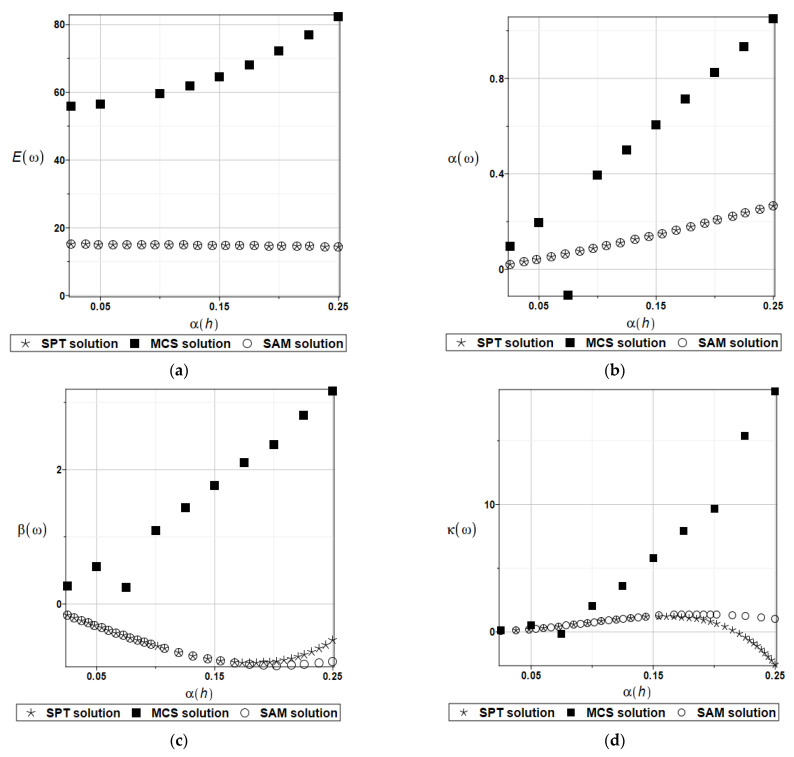
Expected values (**a**), coefficients of variation (**b**), skewness (**c**) and kurtosis (**d**) for a random plate thickness.

Similar to the above, the expected values E(h), coefficients of variation α(h), skewness β(h) and kurtosis κ(h) of the first natural frequency are presented in turn in Figure 9. Additionally, the expected values and coefficients of variation for the first natural frequency are presented in Table 4 and Table 5.

Similar to the previous case, a very good convergence of the results of the SAM and SPT approaches can be observed. The MCS results are in quite good agreement only for kurtosis and α(h) values from 0.0 to about 0.1.

The Monte Carlo simulation technique is the most time-consuming in terms of numerical calculations when performed on a typical PC computer using the MAPLE v.21 computational package. The computation time ratio can be expressed simply by the quotient $\frac{t_{\mathrm{SAM}}}{t_{\mathrm{MCS}}}$ or $\frac{t_{\mathrm{SPT}}}{t_{\mathrm{MCS}}}$, which ranges from 1/50 to 1/100, depending mainly on the number of MCS trials.

## 6. Conclusions

(1) The theoretical and computational studies presented in this work clearly show that the common application of the semi-analytical stochastic approach with continuation methods allows for the fast and accurate determination of the probabilistic coefficients of free vibrations. It is demonstrated that the output randomness in rectangular elastic thin isotropic plate eigenfrequencies is usually not larger than the input statistical scattering of their design parameters. The only exception is in the plate dimension statistics; however, successful measuring techniques are so accurate now that despite the huge sensitivity to this parameter, the realistic coefficient of variation is less than a few percent. The fact that the largest resulting statistical dispersion is obtained for the first eigenfrequency may be important in the reliability assessment of various dynamical systems. This is due to the fact that a limit function, whose probability serves as the basis of the reliability index, is introduced as the difference between the induced vibrations and the lowest eigenfrequency.

(2) A comparative analysis for the first eigenfrequency was performed using three probabilistic approaches: the Semi-Analytical Method (SAM), the Stochastic Perturbation Technique (SPT) and Monte Carlo simulations (MCSs). For the first two methods, results with very high accuracy were obtained. The Monte Carlo simulation showed convergence only for selected random moments—the coefficient of variation for the random plate side length and kurtosis for the random plate thickness.

(3) A continuation of this research can include stochastic extensions of the continuation method in the numerical analysis of forced vibrations, possibly with the use of non-Gaussian random design parameters, too. In the case of any mathematical difficulties with computer algebra integration, the iterative generalized stochastic perturbation technique implemented as the SFEM is recommended. Further uncertainty analysis may be alternatively completed by the application of probabilistic entropy or its relative version presented recently in the literature [17,18].

## Figures and Tables

**Table 1:** Values of the normalized sensitivity gradient for successive design variables on which the value of the first natural frequency of the tested plate depends.

Design Variable Name	Design Variable	Normalized Sensitivity Gradient
Plate length	$l_{x}=l_{y}$ [m]	–1.908109
Thickness of the plate	H [m]	1.136478
Young’s modulus	E [N/m^2^]	0.473791
Poisson ratio	$\nu_{p}$ [–]	0.0669623
Density	$\rho_{p}$ [kg/m^3^]	–0.584767
Stiffness parameter of the damper’s Kelvin element	$k_{0}$ [N/m]	0.0109382
Stiffness parameter of the damper’s Maxwell element	$k_{1}$ [N/m]	–0.0708819
Viscosity parameter of the damper’s Maxwell element	$c_{1}$ [Ns/m]	0.164465
Reference temperature	$T_{0}$ [°C]	0.0189089
Ambient temperature	T [°C]	–0.0295390

**Table 2:** Expected values of the first circular frequency for a random plate side length.

α(b)	E(l)		
	SAM	SPT	MCS
0.025	362.046846366682	362.046846366682	362.047466435600
0.050	362.046908966730	362.046908966730	362.046431550698
0.075	362.047013300146	362.047013300146	362.046932076133
0.100	362.047159366940	362.047159366940	362.046112284009
0.125	362.047347167119	362.047347167119	362.046692036114
0.150	362.047576700696	362.047576700696	362.049805913200
0.175	362.047847967686	362.047847967686	362.048943295710
0.200	362.048160968106	362.048160968106	362.050809955170
0.225	362.048515701978	362.048515701978	362.048477124862
0.250	362.048912169324	362.048912169324	362.051187451775

**Table 3:** Coefficients of variation of the first circular frequency for a random plate side length.

α(b)	α(l)		
	SAM	SPT	MCS
0.025	0.00037913840232059	0.00037913840225768	0.00037820668516146
0.050	0.00075827679791607	0.00075827679779030	0.00075890901680434
0.075	0.00113741518006143	0.00113741517987277	0.00113579512680720
0.100	0.00151655354203154	0.00151655354178001	0.00151584644876523
0.125	0.00189569187710127	0.00189569187678686	0.00189866159120374
0.150	0.00227483017854541	0.00227483017816811	0.00227385578919360
0.175	0.00265396843963868	0.00265396843919851	0.00265356938565002
0.200	0.00303310665365575	0.00303310665315269	0.00303502740231922
0.225	0.00341224481387114	0.00341224481330523	0.00340377236370069
0.250	0.00379138291355934	0.00379138291293050	0.00378941244520895

**Table 4:** Expected values of the first circular frequency for random plate thickness.

α(b)	E(h)		
	SAM	SPT	MCS
0.025	15.1020303938680	15.1020303938679	55.8307084247803
0.050	15.0750528266381	15.0750528266359	56.6189485257520
0.075	15.0309030518086	15.0309030517976	–182.896206992036
0.100	14.9708003252100	14.9708003252100	59.6193578514476
0.125	14.8964516050049	14.8964516049194	61.9339027954595
0.150	14.8100515516881	14.8100515515109	64.6258515558037
0.175	14.7142825280868	14.7142825277585	68.1357033688504
0.200	14.6123145993600	14.6123145988000	72.1942549445554
0.225	14.5078055329993	14.5078055321022	76.9222494795175
0.250	14.4049007988281	14.4049007974609	82.4482857143314

**Table 5:** Coefficients of variation of the first circular frequency for random plate thickness.

α(b)	α(h)		
	SAM	SPT	MCS
0.025	0.021690206735279	0.021690206646010	0.09677329520225
0.050	0.043860569753305	0.043860569571128	0.19522123484153
0.075	0.066951757180877	0.066951756898434	–0.10707504128592
0.100	0.091326253488526	0.091326253094581	0.39417790477171
0.125	0.117231393179819	0.117231392659076	0.49988129568701
0.150	0.144765615806037	0.144765615138953	0.60551075944427
0.175	0.173850845320013	0.173850844482707	0.71407533547204
0.200	0.204216713685934	0.204216712650311	0.82487834596053
0.225	0.235407286078469	0.235407284812828	0.93330578277003
0.250	0.266829037072705	0.266829035543292	1.05206621395503

## Data Availability

Not applicable.
